# The Neurology and Neurosurgery Interest Group (NANSIG)—ten years of cultivating interest in clinical neurosciences

**DOI:** 10.1007/s00701-022-05113-0

**Published:** 2022-01-18

**Authors:** Jay J. Park, Setthasorn Zhi Yang Ooi, Conor S. Gillespie, Soham Bandyopadhyay, Yasir A. Chowdhury, Georgios Solomou, Melissa Gough, Ulrick Sidney Kanmounye, Alvaro Yanez Touzet, Michael T. C. Poon, Andreas K. Demetriades, Michael D. Jenkinson, Alistair Jenkins, Neurology and Neurosurgery Interest Group (NANSIG)

**Affiliations:** 1 Edinburgh Medical School, University of Edinburgh, Edinburgh, UK; 2 Cardiff University School of Medicine, University Hospital of Wales Main Building, Heath Park, Cardiff, UK; 3 Institute of Systems, Molecular and Integrative Biology (ISMIB), University of Liverpool, Biosciences Building, Crown Street, Liverpool, L69 7BE UK; 4 Nuffield Department of Surgical Sciences, Oxford University Global Surgery Group, University of Oxford, Oxford, UK; 5 Department of Neurosurgery, University Hospitals Birmingham, Edgbaston, Birmingham, UK; 6 School of Clinical Medicine, University of Cambridge, Cambridge, UK; 7 Newcastle Upon Tyne Hospitals NHS Foundation Trust, Newcastle, UK; 8 Association of Future African Neurosurgeons, Yaounde, Cameroon; 9 School of Medical Sciences, Faculty of Biology, Medicine and Health, University of Manchester, Manchester, UK; 10 Department of Clinical Neurosciences, Royal Infirmary of Edinburgh, Edinburgh, UK; 11 Department of Neurosurgery, Royal Victoria Infirmary, Newcastle upon Tyne, UK

**Keywords:** Clinical neuroscience, Education, Neurosurgery, Mentorship, Collaboration, NANSIG

## Abstract

Collaboration and successful teamworking are important components of clinical practise, and these skills should be cultivated early in medical school. The breadth of current medical school curricula means that students often have limited exposure to clinical neurosciences. Since its inception in 2009, the Neurology and Neurosurgery Interest Group (NANSIG) has become a national (UK and Republic of Ireland) example of student and junior doctor synergistic collaboration to deliver educational materials, research, conferences, seminars and workshops, as well as advocating for diversity in this field. Recently, it has expanded to incorporate an international audience and cater for a larger group of young medical professionals. The organisation has overcome numerous challenges and is constantly innovating new approaches to harness the necessary knowledge, skills and network to succeed in a career in neurosciences, neurology and neurosurgery. This article summarises the initiatives undertaken by the group over its first 10 years of existence and its organisational structure, as well as its future plans.

**Supplementary Information:**

The online version contains supplementary material available at 10.1007/s00701-022-05113-0.

## Introduction

Neurology and Neurosurgery are some of the most competitive specialties in the United Kingdom (UK) [37], and competition is only likely to stiffen with the implementation of the national neurosurgical workforce plans [39]. This poses a conundrum for medical students, junior trainees and international applicants: how can they best prepare themselves to optimise their chances of becoming a UK neurosurgeon? With limited exposure to neurosurgery and neurosurgeons at medical school [25, 36], students have turned to an external organisation: the Neurology and NeuroSurgery Interest Group (NANSIG) [19].

**Fig. 1:**
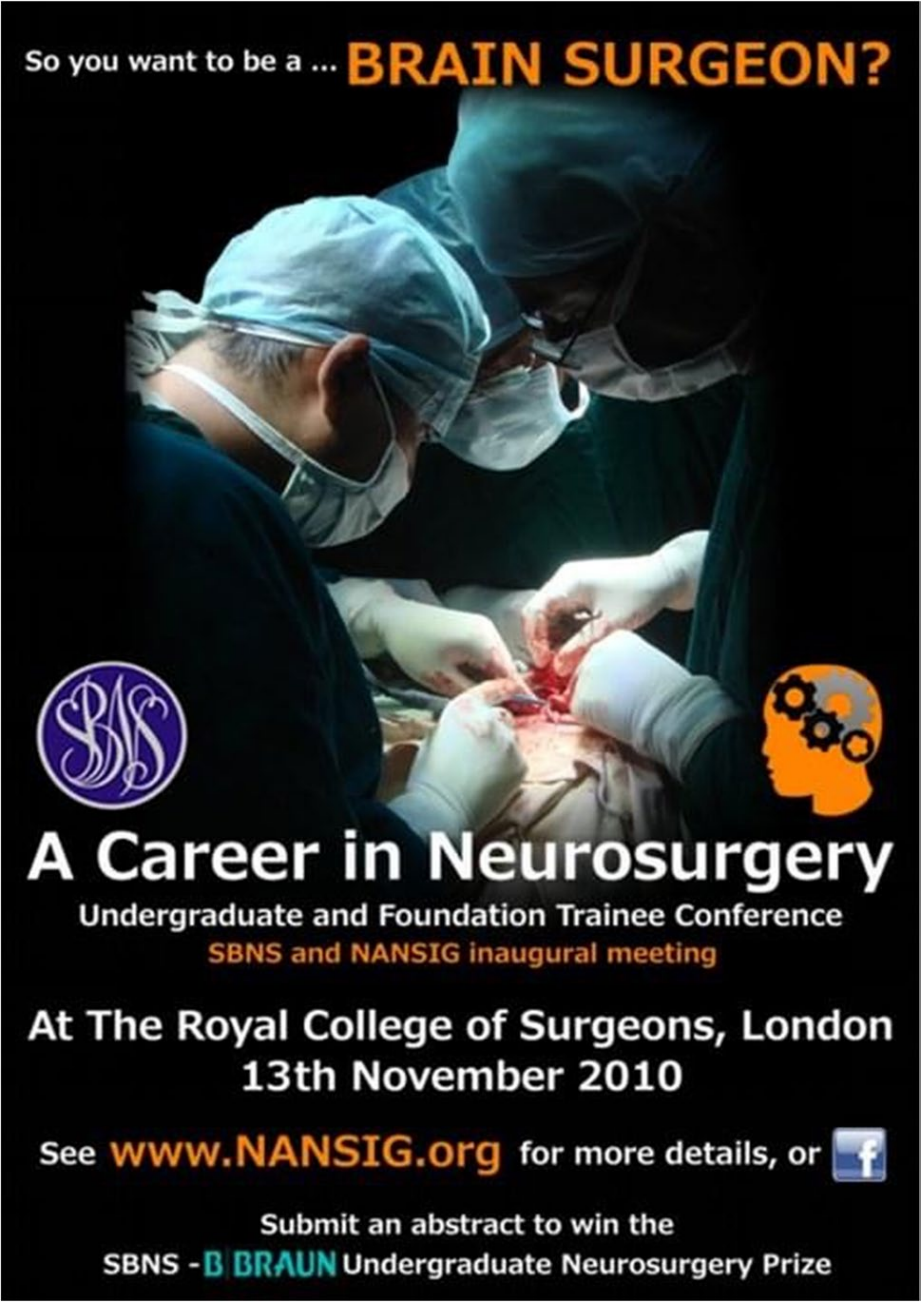
Poster of NANSIG’s inaugural meeting with the Society of British Neurological Surgeons (SBNS)

NANSIG was founded in 2009 by a medical student, after finding that nearly two-thirds of medical students had no exposure to neurosurgery by the end of medical training [40]. The aim of NANSIG was to demystify a career in the clinical neurosciences, and provide students and doctors with tools to become successful neurosurgical trainee applicants. NANSIG’s inaugural meeting was held at the Society of British Neurological Surgeons (SBNS) meeting in London, UK, on the 13th of November 2010 (Fig. 1). Nearly 200 prospective neurosurgeons attended, and an official relationship with the SBNS was created [41]. Since then, SBNS has recognised NANSIG on its official website [32], as an organisation that represents the interests of students and foundation trainees who wish to explore and promote the field of clinical neuroscience. In addition, the SBNS provides NANSIG members discounts for their events, as well as logistics support for the events we organise. These gave NANSIG the resources and publicity it needed to propel itself forward from inception to becoming the organisation it is today.

**Fig. 2:**
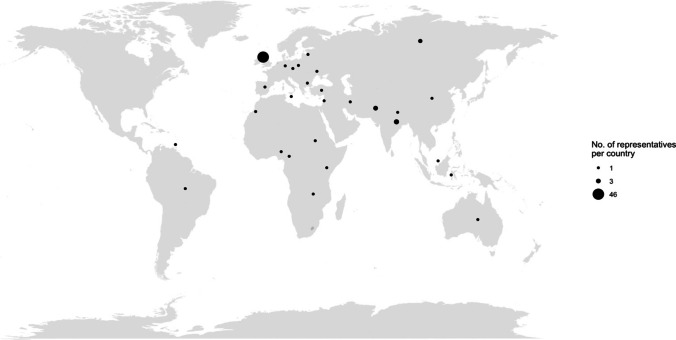
Bubble map of the location of NANSIG representatives worldwide. Bubble sizes correlate to the number of representatives in each country

In the 10 years that have followed, NANSIG has proliferated into a leading student and junior doctor interest group, with over 1800 members, 18 core committee members, 13 Foundation School representatives (deanery leads) [15], 32 medical school representatives (university leads) [27] and 36 international ambassadors representing 30 countries (Fig. 2) (Supplementary table 1). In addition to setting up a national network, NANSIG has completed several national multi-centre studies, and driven neuroscience engagement and progression. With the long-standing support of the SBNS [20, 23], the Association of British Neurologists (ABN) and the British Neurosurgical Trainee Research Collaborative (BNTRC), NANSIG has become the UK’s premier student neuroscience collaborative organisation. Events are held regularly throughout the year including an annual neurosurgery careers day [19], neurosurgical skills workshops [20, 23] and educational and advocacy courses [6, 28, 33]. We have also supported the annual ABN Student Days to promote research and networking opportunities for students and trainees interested in neurology [1, 2, 31].

### Organisational structure

**Fig. 3:**
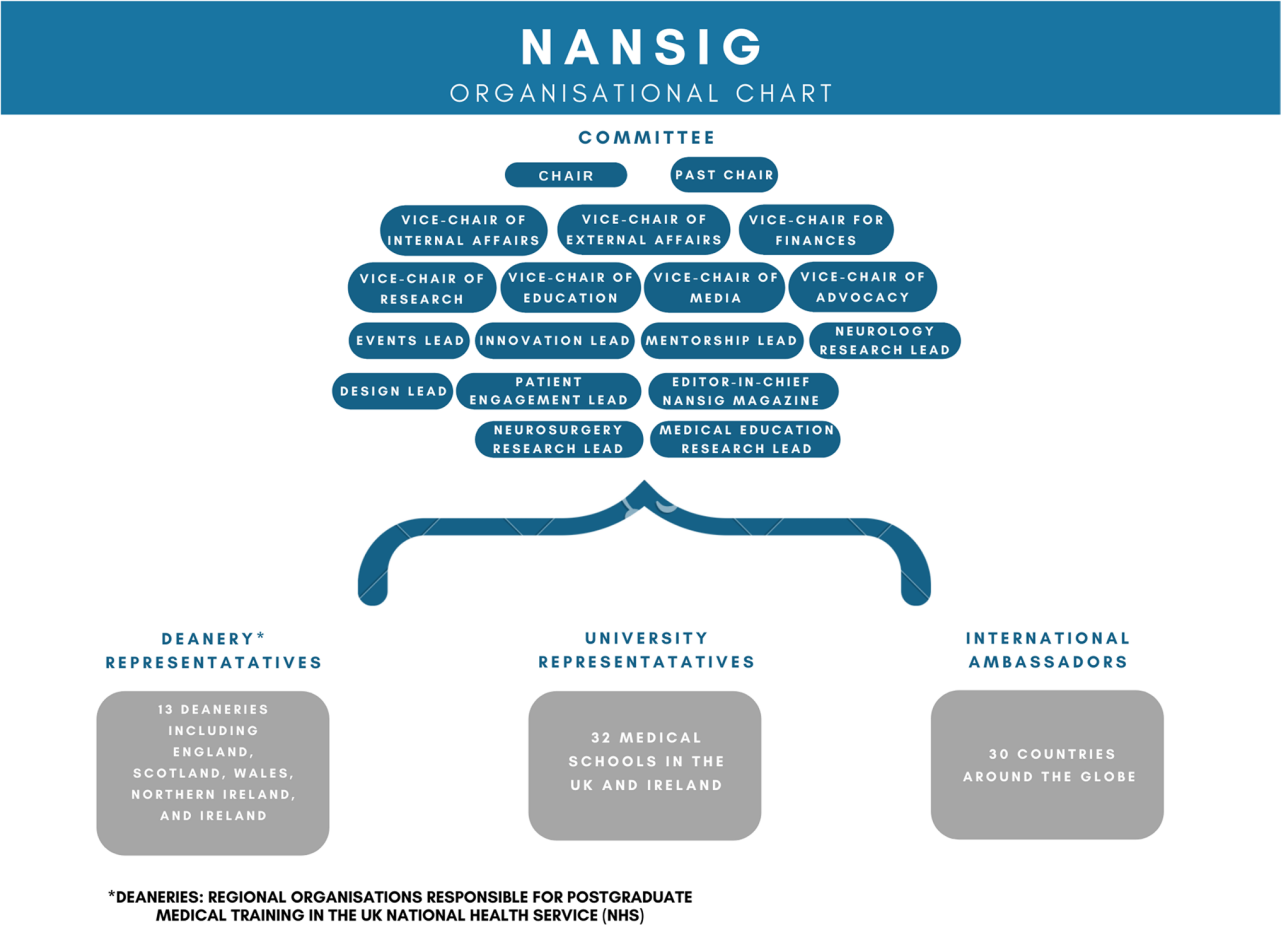
NANSIG organisational chart

NANSIG consists of a core committee, regional leads (consisting of university and deanery leads), international ambassadors and its members (Fig. 3). List of deaneries, universities and countries are listed in Supplementary table 1, and the specific roles of our committees are explained in Supplementary table 2. This structure has developed over time and is constantly evolving to maximise efficiency in executing projects, and to provide equal opportunities to all medical students and junior doctors. NANSIG’s membership is free and is defined by an individual’s subscription to the monthly newsletter on the NANSIG website (nansig.org). Currently, NANSIG has more than 1800 members worldwide, with over 50% actively engaging every month, making it one of the biggest global student-led organisations with an interest in clinical neurosciences.

All members are eligible to apply to become core committee members, regional leads or international ambassadors. Elections for these positions occur annually with the term of each role being 1 year: in January for the committee, and in August for leads and ambassadors. NANSIG is committed to organising all elections in accordance with the diversity principles of the ALBA network, relating to consistency in selection criteria, and minimising bias when possible [3, 38]. This is to ensure that candidates are elected solely based on their suitability for the role and their ability to contribute to the clinical neuroscience community.

### Research

In the past 4 years, NANSIG has established itself as a platform for members to collaborate and contribute to high-quality multi-centre research; some of which has informed national specialty reports [16, 21]. To date, NANSIG has been involved in seven (three complete, four ongoing) national research, audit and quality improvement projects, with several in the process of being developed (Table 1). The project themes are uniquely conceptualised by students or junior doctors in the NANSIG core committee. Many of NANSIG’s past and ongoing projects have received the support from SBNS Academic Committee and theBNTRC in the form of project supervision and guidance, protocol review and identifying members of the data collaboration team.

**Table 1:** Table of NANSIG research projects

Study name	Description	Project start	Project end	No. of collaborators	No. of students/foundation doctors	No. of medical schools/units	No. of patients/participants	No. of conference presentations	No. of publications
ENTICE [16]	Retrospective study assessing adherence of cauda equina syndrome referrals to published national guidelines. Supported by the BNTRC	2016	2017	86	56	28	4441	2	1
STUN [14]	Cross-sectional study of undergraduate neuroanatomy teaching in the UK and Ireland	2019	2020	11	6	24	N/A	1	1
ELISAR-GB	Prospective evaluation of intraoperative surgical adjuncts used in the resection of glioblastoma tumours	2019	Ongoing	84	46	28	291	2	-
SPICE-19 [5, 8, 34]	Prospective observational study of mental health and well-being of medical students and foundation doctors during the COVID-19 pandemic	2020	2020	35	34	32	2075	10	4
CRANIAL [9, 10, 24]	Prospective observational cohort study of cerebrospinal fluid rhinorrhoea rates after endonasal intervention to the skull base	2020	Ongoing	148	34	30	869	5	3
National Evaluation of Clinical Neuroscience Teaching at UK Medical Schools	Cross-sectional study of undergraduate clinical neuroscience teaching in the UK	2020	Ongoing	7	7	35	N/A	-	-
SPICE-20	Prospective observational study of mental health and well-being of medical students and foundation doctors during the COVID-19 pandemic	2021	Ongoing	39	38	33	780	-	-
U-BECTS	Retrospective audit of the clinical management and outcomes of patients with Benign Epilepsy with Centro-Temporal Spikes (BECTS)	2021	Ongoing	Inviting collaborators		Inviting units	-	-	-
NAPIER	Retrospective audit of pathways in epilpetic seizure referrals	2021	Ongoing	Inviting collaborators		Inviting units	-	-	-
INTERVAL-GB	Retrospective audit of MRI surveillance practice in the UK and Ireland after surgery for patients with glioblastoma	2021	Ongoing	Inviting collaborators		Inviting units	-	2	-
TOP-TBI Mini Project	Prospective cohort study on timing of pharmacological prophylaxis for venous thromboembolism in patients with traumatic brain injury	2021	Ongoing	Inviting collaborators		Inviting units	-	-	-

Furthermore, NANSIG has established a cross-continental collaboration with the Association of Future African Neurosurgeons (AFAN) to develop a research incubator programme, the first of its kind in the global neurosurgical community. This programme provides students and junior doctors with the opportunity to design and lead global neurosurgical research projects within a topic of their interest, under the mentorship of experienced academics. Though in its infancy, the collaborative has since contributed to the scholarly output in Africa through identifying areas in need of improvement, such as the lack of availability and accessibility of diagnostic and management tools for aneurysmal subarachnoid haemorrhage in the continent [12, 13, 35].

Alongside these collaborative studies, NANSIG has also published many articles discussing a range of topics including (i) women in neurosurgery and advocacy [6, 28, 33], (ii) medical education [26], (iii) neurosurgical training in the UK [29, 37], and (iv) neurosurgical skills development [20, 23]. Together, these have accumulated over 26,500 views and 84 citations to date. A total of 22 students and foundation trainees have also gained first authorship from our published work. These projects have provided many of NANSIG’s members the opportunity to develop key research skills, such as study development, data analysis, critical appraisal and manuscript writing. In the past 5 years, 13 members of NANSIG’s core committee have gone on to become neurosurgical trainees, with 8 of those gaining highly competitive academic registrar appointments.

### Education

With NANSIG tackling barriers that medical students and junior doctors face pursuing a career in the clinical neurosciences, an educational component emerged to counter neurophobia [22] and promote enthusiasm [17]. To ensure that financial or geographic constraints did not limit access, all educational resources were uploaded onto open platforms. For example, videos on pathophysiology, diagnosis and management of common neurosurgical and neurological pathologies were uploaded to YouTube. Over 50 videos are available that have accumulated over 60,000 views worldwide [30]. NANSIG members interested in medical education voluntarily prepared contents for these videos, which were then validated by a consultant neurologist or neurosurgeon. This resource serves as a learning opportunity for students and junior doctors, whilst providing tutors with the opportunity to deliver teaching on a specific neurological topic of their interest. It is our aim to create a peer-learning environment, as the process of both teaching and learning is a pivotal experience in becoming a competent physician [11].

In keeping with international trends through the COVID-19 pandemic, since March 2020, the scope of the educational content has expanded to include a webinar series on neuroanatomy, common conditions encountered in different clinical neuroscience sub-specialties and a journal club. This branch of educational activity has been solely developed and delivered by leading academic clinicians, including world experts on a topic or principal investigators of practise-changing research (Table 2).

**Table 2:** List of NANSIG educational initiatives

Program name	Description	Duration	Platform
Revision Videos	Basic neurology and neurosurgical education series are produced by students and foundation year doctors. Each video is validated by a neurosurgeon or a neurologist	2017–current	YouTube
Neurosurgical Skills Workshop	Workshop with key neurosurgical skills, including proning and pinning, burr holes, drain insertion, suturing and more. Now in its 5th annual edition	2018–current	In-person
COVID-19 Podcast	Neurologists and neurosurgeons were interviewed and asked for their personal insight into the COVID-19 pandemic. Podcasts were then uploaded to NANSIG.org	2020	Website
Journal Club	Principal investigators of a practice-changing research paper detailing the steps involved in developing, delivering and reporting their studies: ULTRA trial, ROAM trial, CRASH, Dex-CSDH	2018–current	Virtual
Webinars	World experts discuss common cases and dilemmas faced in their subspecialty: spinal cord injury, neuro-oncology, functional neurosurgery, skull base neurosurgery, paediatric neurosurgery and artificial intelligence in neurosurgery	2017–current	Virtual
Patient Leaflet	Detailed and terminology friendly leaflets were created to aid patients in understanding either a neurological condition or procedure	2020–current	Website
Instagram Cases	Instagram posts detailing case vignettes that allow readers to solve and consider possible causes, diagnosis and management plans	2020–2021	Instagram
Neuro-Cheat Sheets	Fact-files created for students to aid in revision and individual study	August 2021	Website

Since 2015, NANSIG has also hosted six in-person and two virtual neurosurgical workshops. Workshops comprise a structured core curriculum to support acquisition of essential skills required of a junior neurosurgical trainee. These include positioning and pinning, drilling burr holes, acquiring ventricular access and performing craniotomies. The delivery of these skills is achieved through teaching delegates the fundamentals of neuroanatomical knowledge and microsurgical equipment handling.

These learning opportunities are not provided in the traditional curricula of medical schools [23] nor are they easily accessible during the UK Foundation Programme. The low student-to-teacher ratio (2:1) facilitates an optimum training experience delivered by the faculty of consultants and senior neurosurgical registrars; these courses have been proven to enhance surgical skills [20, 23]. The positive reception to these workshops and unanimously positive reviews have led to workshops being oversubscribed with the most recently completed—the External Ventricular Drain (EVD) Simulation Workshop—receiving over 400 applications for attendance worldwide.

### Conference and networking

The annual NANSIG Neurosurgery Careers Day is organised in collaboration with the SBNS. Each year, the event attracts hundreds of delegates, from across the UK and internationally. The 10^th^ and most recent Careers Day was a virtual event, and catered to over 230 attendees and presenters from 30 countries across the globe [19].

The Careers Day invites trainees, consultants and professors in neurosurgery to deliver keynote speeches on a variety of topics such as preparing for a career in academic neurosurgery, insights into a career in neurosurgery and global neurosurgery. These sessions which include dedicated, individual advice from a trainee as part of ‘Neurosurgery CV clinics’ and inter-person networking events also provide delegates with the opportunity to network and build lifelong working relationships with trainees and neurosurgeons, which may lead to fruitful collaborations in the future. The presentation of research abstracts, which have featured oral presentations, and ‘flash’ poster presentations, also provides delegates with the opportunity to develop presentation, time management skills and creative, lateral thinking.

### Mentorship

In January 2021, NANSIG’s mentorship pilot scheme was offered to regional leads. In total, 18 neurologists and neurosurgeons were recruited as mentors, and 31 mentees were matched to a respective mentor according to individual interests. Progress of the scheme was monitored through constant feedback, which was unanimously positive. With the pilot scheme proving to be in great demand, the official mentorship programme was launched in September 2021. The scheme received over 250 applications from prospective mentees, with the scheme due to commence at the end of 2021.

### Advocacy

NANSIG first began its “Neurosurgeon of the Month” advocacy campaign on social media in April 2020. A key focus of this initiative was to recognise inspirational past and present female neurosurgeons to highlight role models and mentors, both of which are key motivating factors to encourage and retain women in the specialty [28]. A written post and image of the selected neurosurgeon was shared on NANSIG’s newsletter to members worldwide and on social media to the public. The campaign has reached over tens of thousands of individuals globally across NANSIG platforms, and NANSIG has seen a substantial increase in the number of core committee members that identify as female in 2021 compared to 2019 (35% vs 18%), with many citing the campaign as a motivating factor to apply for the role. In addition, 55.6% of the current core committee are from an ethnic minority group.

### Communications

NANSIG aims to employ effective communication via social media and technology. Our official website, nansig.org, provides an abundance of resources for students, junior doctors and healthcare professionals interested in the field of neurology and neurosurgery, and received 2625 visits per month in 2021.

In addition to our website, we actively host social media platforms via Facebook (> 3100 likes), Twitter (@nansig1, > 2000 followers and 26,023 impressions on average each month from January–July 2021), Instagram (@nansig_group, > 800 followers), Linkedin and YouTube (@nansig, > 1100 subscribers). Through these diverse channels, we raise awareness of our events and causes and provide opportunities to engage with interested individuals.

We communicate with all members of NANSIG through subscribed mailing lists [7]. In these communications, we have monthly newsletters distributed through the mailing list to communicate news and opportunities to members.

### Challenges

NANSIG has overcome several challenges since its establishment. First, one of the studies that was started but not completed was the National Traumatic Brain Injuries Referrals study in 2018. This project encountered difficulty with centre recruitment, given that there was an existing national trauma network already in place. Learning from this, we have implemented an important process when planning and constructing the protocol of a study, which is to consult experienced researchers from organisations such as the BNTRC and SBNS for advice and support throughout.

Second, based on feedback from our Annual Careers days, it was evident that the event was not fully inclusive. Since then, we have tried to increase inclusivity and diversity within all our events, such as by organising our first-ever advocacy conference to educate and inspire members, creating a core committee vice chair of advocacy role to encourage representation and internally auditing the diversity perceptions of all NANSIG events [4]. This involves seeking feedback from delegates for multiple speaker (‘panel’) events, on the representation of the speakers. Furthermore, we have taken an active stance in raising awareness and addressing the issue of gender inequality in this field through research and publications [6, 28, 33]. One of our core efforts in bridging the gap of women in neurosurgery is through our advocacy programme [28].

Third, another issue identified was the need to have a standardised and clear approach to authorship of publications, and this is communicated from inception. This is now clearly defined in all research protocols.

Fourth, due to the structure of the organisation and relative interest from our members, the field of Neurology has been a lesser component of NANSIG’s focus and initiatives. With the launch of two neurology-themed multi-centre collaborative studies and an inaugural NANSIG Neurology Careers Day, we hope to broaden our scope of events, research and projects to ensure NANSIG provides useful resources to promote clinical neurosciences, in addition to Neurosurgery.

Finally, NANSIG has been privileged to experience such an accelerated expansion. In a single year, we had an increase from 1000 members to over 1800 members, whilst the committee expanded from ten members to 18. We have also started actively engaging with our international ambassador programme, in which we newly recruited ambassadors from 30 different countries. This had put a substantial amount of pressure on the Chair and the core committee. Such pressures are expected and are a promising measure of NANSIG’s utility 10 years into its establishment.

## Future plans

NANSIG’s future vision can be dichotomised into improving existing initiatives, and championing new initiatives to help promote a career in clinical neurosciences. We hope to maintain a strong relationship with both the SBNS and ABN, as well as newer partner organisations like the European Association of Neurosurgical Societies (EANS), the British Paediatric Neurology Association (BPNA), BNTRC and AFAN. We will continue to provide high-quality education and mentorship and to serve as a platform for those interested in the clinical neurosciences to express and develop their interest on a national and international scale. We will progress our existing body of research by learning from our past experiences, incorporating a proactive approach emphasising clarity and project preparation and seeking guidance throughout the entirety of the research process, from the expert bodies we are associated with. We hope this will lead to constructing an academic framework to facilitate constructive academic guidance from senior collaborators.

Clarity, openness and fairness are at the heart of NANSIG’s vision going forward. This has been tackled through (1) an official NANSIG constitutional document—(nansig.org/constitution); (2) a clear, standardised and fair approach towards the election of committee members and research authorship (nansig.org/policy); (3) turning our attention to advocacy; and (4) encouraging participation in line with the ALBA framework. We also hope to involve patients, carers and families in future research projects.

Finally, the prospect of collaborating on a global level is exciting. NANSIG is now well known internationally—this is indicative of our plan to expand, increasing representation on a global scale. We hope that the NANSIG model is an exemplary story that can assist the development of similar neurology/neurosurgery interest groups in other countries, for the ultimate benefit of improving the quality of care for patients.

## Conclusion

In the 10 years since its formation, NANSIG has become much more than a group of interested medical students. We have evolved to be a pioneering organisation that delivers collaborative research and education, whilst developing the next generation of neurologists, neurosurgeons and neuroscientists, and advocating for inclusivity within the specialty. Nevertheless, we believe that there is still great potential for development and growth. We aim to continue striving to promote awareness and participation in the clinical neurosciences, and encourage successful collaborations—a cornerstone of the senior organisations that support us.

## Supplementary Information

Below is the link to the electronic supplementary material.

Supplementary file1 (DOCX 20 KB)
