# Supplementary material for: The Neurology and Neurosurgery Interest Group (NANSIG)—ten years of cultivating interest in clinical neurosciences
Source: Acta Neurochir (Wien). 2022 Jan 18;164(4):937–46. doi: 10.1007/s00701-022-05113-0 (PMC8763620; doi:10.1007/s00701-022-05113-0)
Supplement: Supplementary file 1 — Supplementary file1 (DOCX 20 KB) [file 701_2022_5113_MOESM1_ESM.docx]

Supplementary Table 1. List of medical schools, deaneries and countries which have NANSIG representatives for the academic year 2021/22. This list is accurate as of 14th October 2021.

| **University Leads** | **Deanery Leads** | **International Ambassadors** |
| --- | --- | --- |
| Aston University Medical School | East Midlands | Australia |
| University of Aberdeen School of Medicine and Dentistry | Eastern | Brazil |
| Barts and The London School of Medicine and Dentistry | London - North East Thames | Bulgaria |
| University of Birmingham College of Medical and Dental Sciences | London - North West Thames | Cameroon |
| Brighton and Sussex Medical School | North Western | China |
| University of Bristol Medical School | Oxford | Congo, The Democratic Republic |
| University of Buckingham Medical School | Peninsula | Czech Republic |
| University of Cambridge School of Clinical Medicine | Republic of Ireland | Germany |
| Cardiff University School of Medicine | Scotland - East of Scotland | Ghana |
| University of Dundee School of Medicine | Scotland - West of Scotland | Grenada |
| The University of Edinburgh Medical School | West Midlands | India |
| University of Exeter Medical School | Yorkshire | Indonesia |
| University of Glasgow School of Medicine |  | Iran |
| Keele University School of Medicine |  | Kenya |
| King's College London GKT School of Medical Education |  | Latvia |
| University of Leeds School of Medicine |  | Lebanon |
| University of Limerick |  | Malaysia |
| University of Liverpool School of Medicine |  | Malta |
| University of Manchester Medical School |  | Morocco |
| National University of Ireland Galway |  | Nepal |
| Newcastle University School of Medical Education |  | Nigeria |
| University of Nottingham School of Medicine |  | Pakistan |
| University of Oxford Medical Sciences Division |  | Poland |
| Queen's University Belfast School of Medicine |  | Russia |
| Royal College of Surgeons Ireland |  | Spain |
| University of Sheffield Medical School |  | State of Palestine |
| University of St Andrews School of Medicine |  | Sudan |
| St George's, University of London |  | Turkey |
| Trinity College Dublin |  | Ukraine |
| University College London Medical School |  | Zambia |
| University of Central Lancashire School of Medicine |  |  |
| University of Warwick Medical School |  |  |

Supplementary table 2. NANSIG Core committee roles (correct as of 07/11/2021).

| **NANSIG Committee Roles** | |
| --- | --- |
| Chair | Responsible for making executive decisions, and coordinating the management, leadership, and direction of NANSIG. |
| Vice-Chair of Advocacy | Ensures the representation of clinicians and academics from diverse backgrounds in all our events and organises further initiatives to promote advocacy. |
| Vice-Chair of Education | Organises educational opportunities including webinars and materials for medical students, junior doctors, and members who are interested in the field of clinical neurosciences. |
| Vice-Chair of External Affairs | Managing and leading international ambassadors in engaging with NANSIG through events, research, and education. |
| Vice-Chair of Finances | Responsible for managing NANSIG’s ledger, acquiring sponsorships, allocating budgets for conferences, workshops, talks, and research. |
| Vice- Chair of Internal Affairs | ‘Day to day’ communication with regional leads, deanery leads, design and distribution of NANSIG newsletter. |
| Vice-Chair of Media | Website and social media maintenance, post and create event pages on social media. |
| Vice-Chair of Research | Coordinate all research initiatives and existing projects. |
| Design Lead | Promotional materials, banners, flyers, and abstract design for Neurosurgery careers day, workshops, and all educational events. |
| Editor-In-Chief of NANSIG Magazine | Production of the bi-monthly NANSIG magazine. |
| Events Lead | Responsible for the organisation of the Neurosurgery careers day, and Neurosurgical Skills workshop. |
| Innovation Lead | Optimise NANSIG platforms and technology to facilitate research, and educational initiatives. |
| Medical Education Research Lead | Organisation of medical education research projects. |
| Mentorship Lead | Directing the NANSIG Mentorship programme. |
| Neurology Research Lead | Coordinate and directly lead neurology based research projects. |
| Neurosurgery Research Lead | Coordinate and directly lead neurosurgery based research projects. |
| Past Chair | Executive position for previous chair- offer advice, mentorship and guidance to all current committee members. |
| Patient Engagement Lead | Producing and coordinating patient information leaflets. |
